# Supplementary material for: Insights into substrate binding and utilization by hyaluronan synthase
Source: bioRxiv. 2025 Oct 18:2025.10.17.683186. Preprint. [Version 1] doi: 10.1101/2025.10.17.683186 (PMC12632751; doi:10.1101/2025.10.17.683186)
Supplement: 1 [file NIHPP2025.10.17.683186V1-supplement-1.pdf]

bioRxiv preprint doi: https://doi.org/10.1101/2025.10.17.683186; this version posted October 18, 2025. The copyright holder for this preprint (which was not certified by peer review) is the author/funder, who has granted bioRxiv a license to display the preprint in perpetuity. It is made available under aCC-BY 4.0 International license.

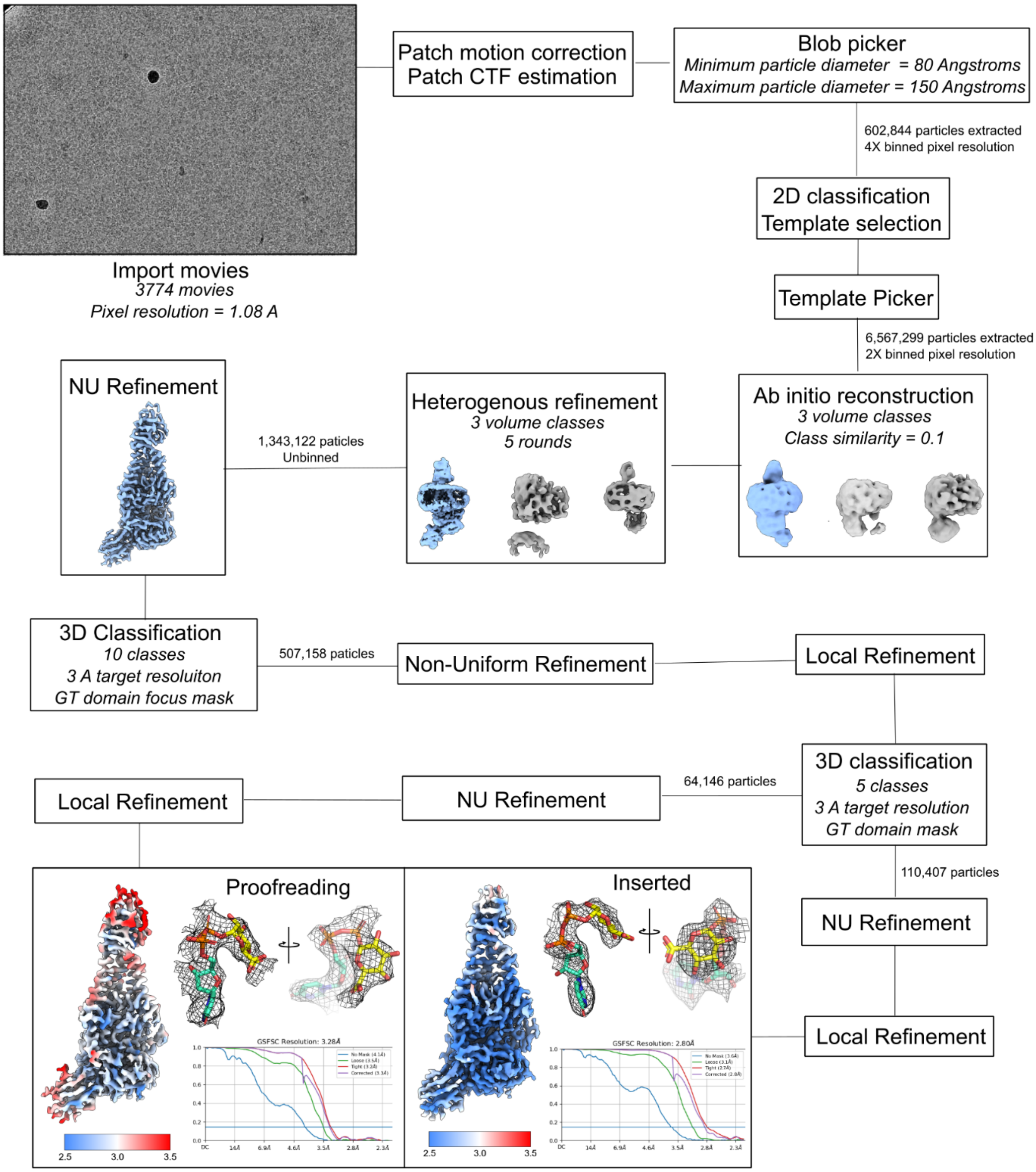

Supplementary Figure 1: CryoEM data processing for UDP-GlcA inserted and proofreading structures. Carves of cryo electron density maps for UDP-GlcA in inserted and proofreading states are displayed as a black mesh. Local resolution maps are colored according to estimated resolution at FSC = 0.143 in Å.

bioRxiv preprint doi: https://doi.org/10.1101/2025.10.17.683186; this version posted October 18, 2025. The copyright holder for this preprint (which was not certified by peer review) is the author/funder, who has granted bioRxiv a license to display the preprint in perpetuity. It is made available under aCC-BY 4.0 International license.

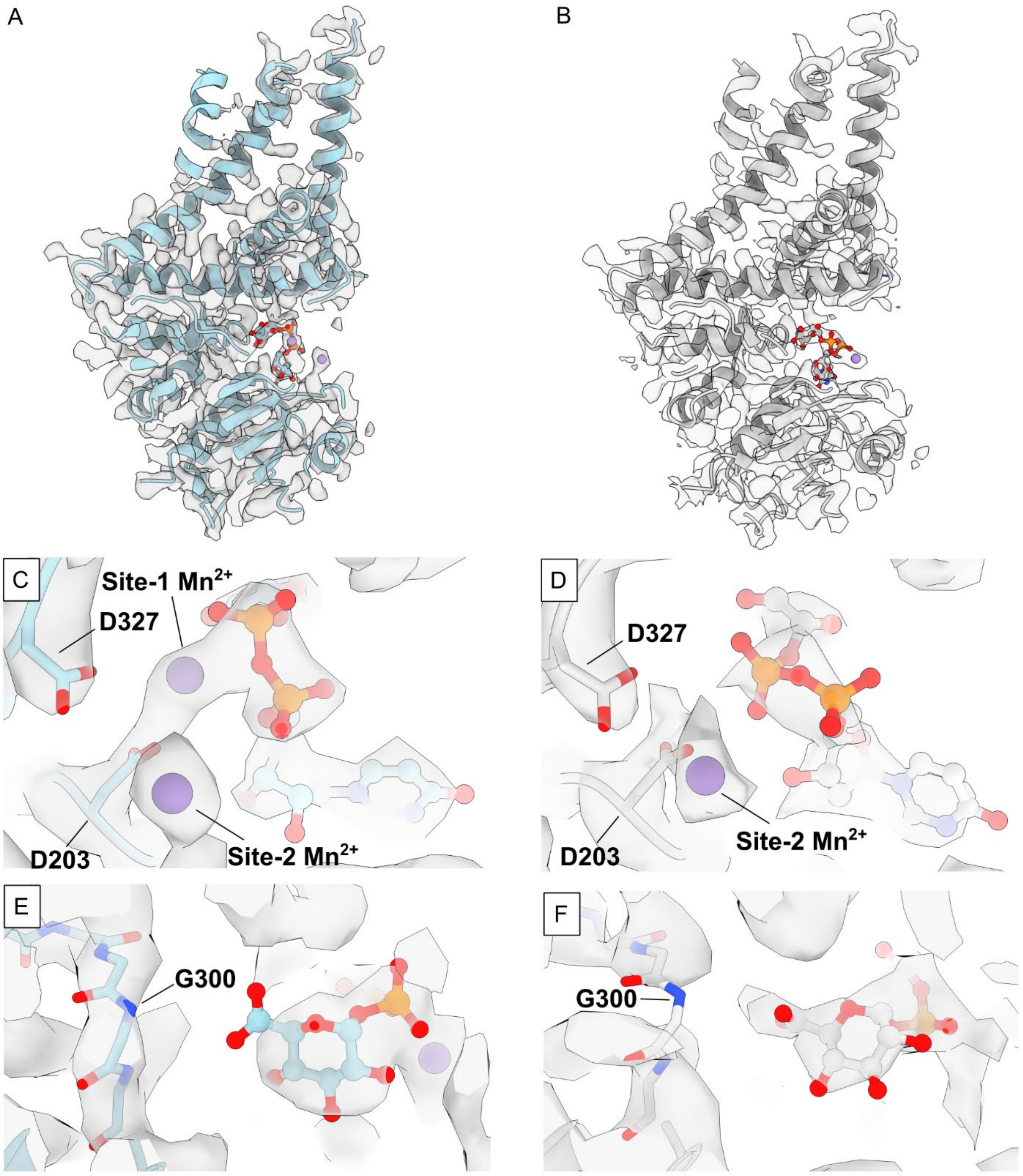

Supplementary Figure 2: CryoEM density for Mn and the priming loop c-terminus in inserted and proofreading conformations. A, Cross-section of the cryoEM density map for the inserted UDP-GlcA pose. CvHAS and ligand carbon atoms are colored in light cyan, with the ligand represented as a ball and stick model. B, Cross-section of cryoEM density map for the proofreading UDP-GlcA pose. Protein and ligand carbon atoms are colored in

bioRxiv preprint doi: https://doi.org/10.1101/2025.10.17.683186; this version posted October 18, 2025. The copyright holder for this preprint (which was not certified by peer review) is the author/funder, who has granted bioRxiv a license to display the preprint in perpetuity. It is made available under aCC-BY 4.0 International license.

grey. C-D Active site view showing local cryoEM density for putative manganese ions (purple) in inserted (C) and proofreading (D) UDP-GlcA bound CvHAS. E, Active site view showing continuous cryoEM density for Gly300 in the inserted UDP-GlcA bound state. F, Broken cryoEM density due to an unresolved Gly300 in the proofreading UDP-GlcA bound state.

bioRxiv preprint doi: https://doi.org/10.1101/2025.10.17.683186; this version posted October 18, 2025. The copyright holder for this preprint (which was not certified by peer review) is the author/funder, who has granted bioRxiv a license to display the preprint in perpetuity. It is made available under aCC-BY 4.0 International license.

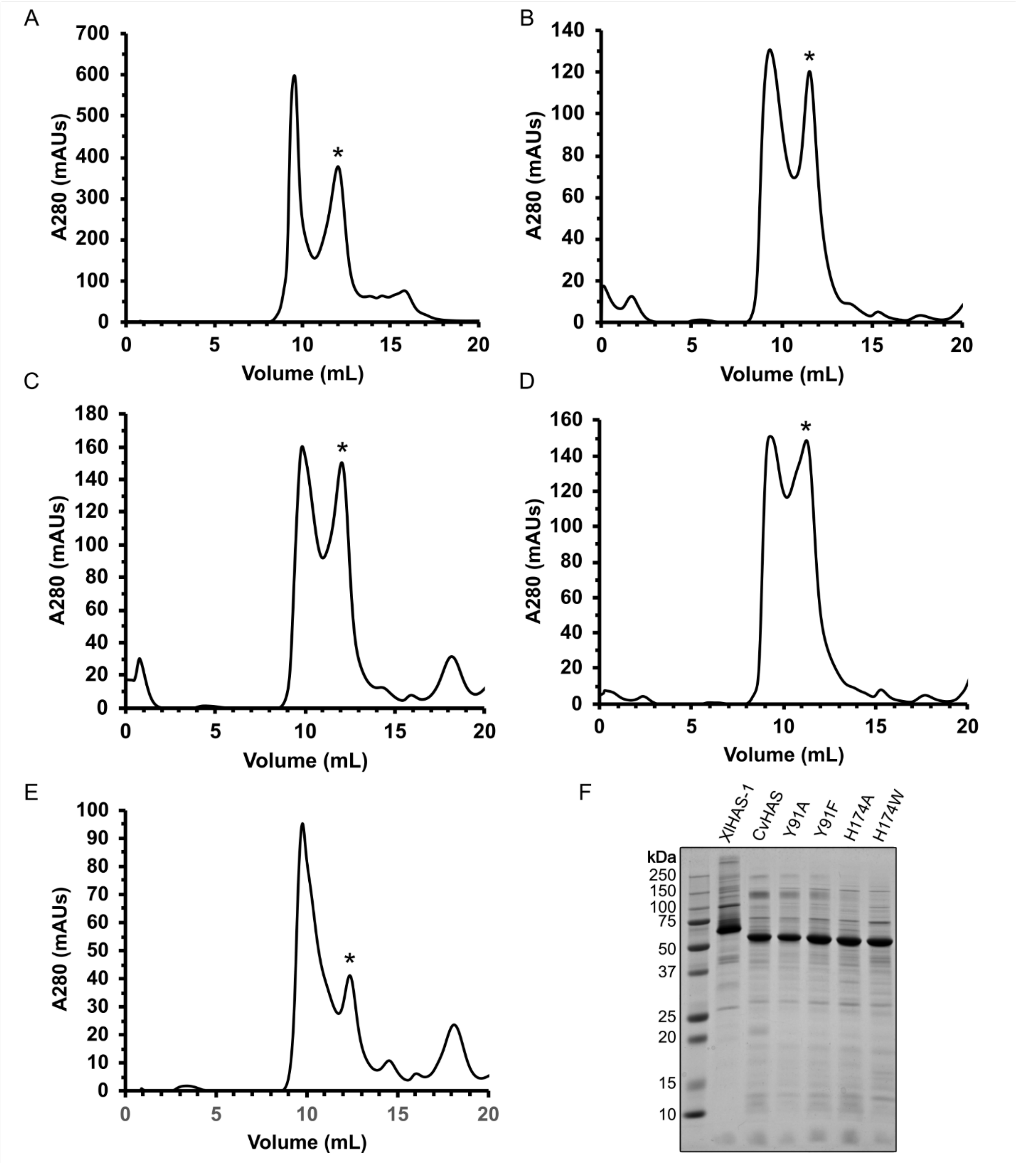

Supplementary Figure 3: Purification CvHAS' uracil pocket mutants. A-E, S200 Increase chromatography of WT CvHAS (A), Y91A (B), Y91F (C), H174A (D) and H174W (E). Peaks pooled for subsequent biochemistry are indicated with an asterisk (*). F, Coomassie stained SDS-PAGE gel for purified XlHAS-1, WT CvHAS, and CvHAS mutants.

bioRxiv preprint doi: https://doi.org/10.1101/2025.10.17.683186; this version posted October 18, 2025. The copyright holder for this preprint (which was not certified by peer review) is the author/funder, who has granted bioRxiv a license to display the preprint in perpetuity. It is made available under aCC-BY 4.0 International license.

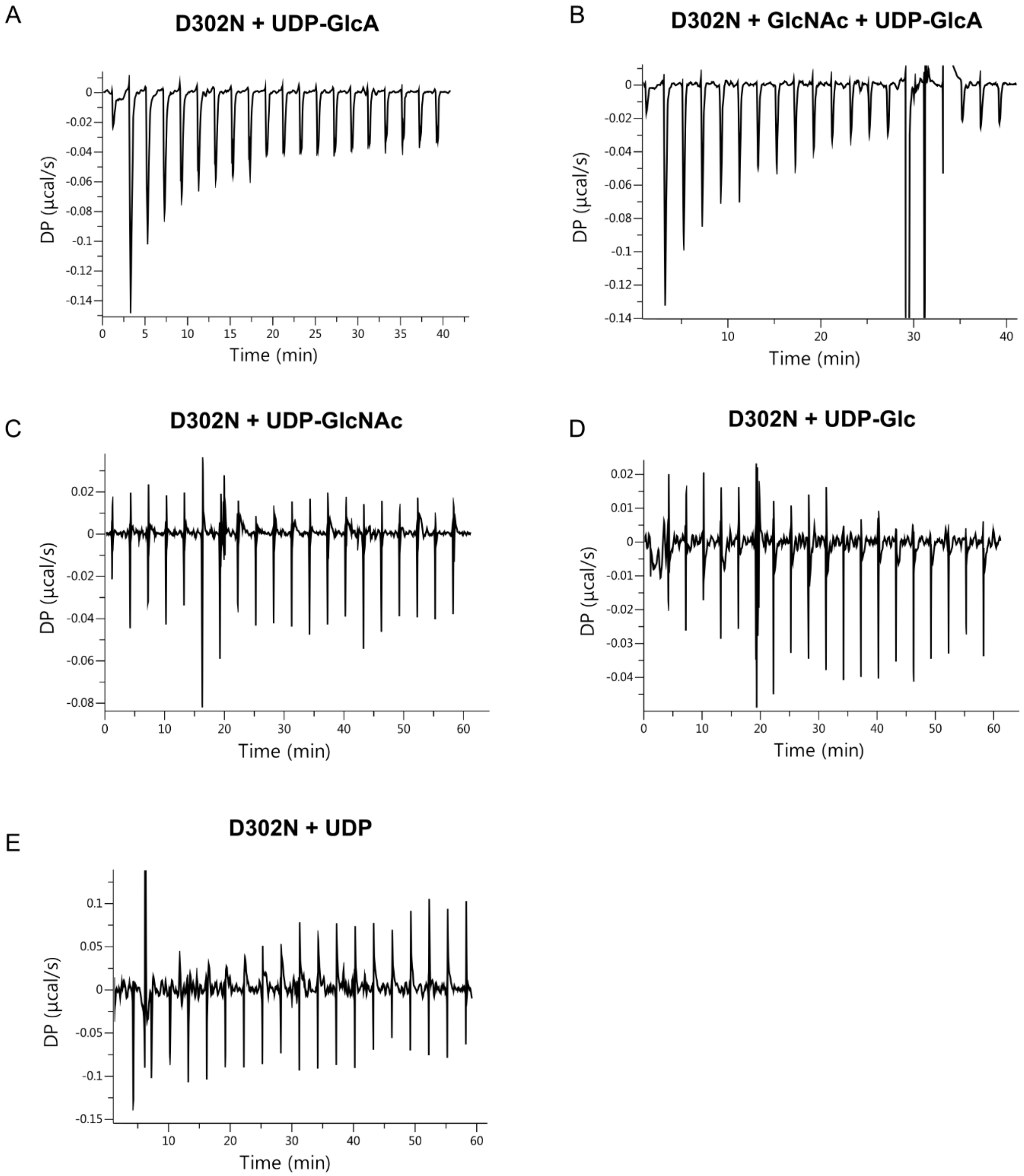

Supplementary Figure 4: ITC plots for CvHAS substrate titration. A-B, raw ITC plots for UDP-GlcA titration in the absence (A) or presence (B) of excess GlcNAc. C-E, Raw ITC plots for UDP-GlcNAc, UDP-Glc and UDP.

bioRxiv preprint doi: https://doi.org/10.1101/2025.10.17.683186; this version posted October 18, 2025. The copyright holder for this preprint (which was not certified by peer review) is the author/funder, who has granted bioRxiv a license to display the preprint in perpetuity. It is made available under aCC-BY 4.0 International license.

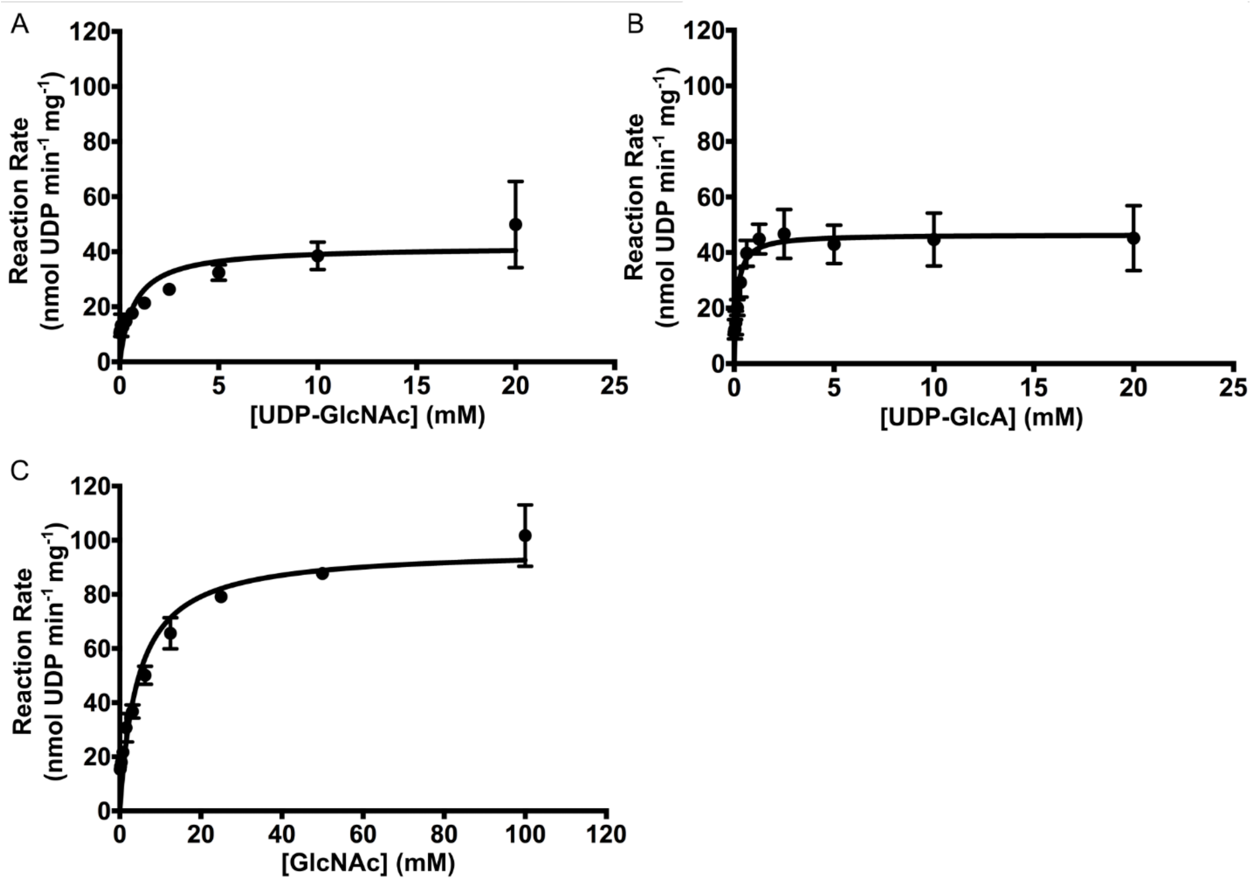

Supplementary Figure 5: Michaelis-Menten fits for CvHAS substrate titration. A, CvHAS titration with UDP-GlcNAc in the absence of an acceptor. B, Titration of UDP-GlcA in the presence of 10 mM GlcNAc. C, Titration of GlcNAc in the presence of 2.0 mM UDP-GlcA. Five technical replicates were used to derive average reaction rates and standard deviations for plotting of UDP-GlcNAc and UDP-GlcA titrations. Three technical replicates were used for fitting the GlcNAc titration series. All non-linear regressions were performed in Prism 6.0. Error bars represent standard deviations from the means.

bioRxiv preprint doi: https://doi.org/10.1101/2025.10.17.683186; this version posted October 18, 2025. The copyright holder for this preprint (which was not certified by peer review) is the author/funder, who has granted bioRxiv a license to display the preprint in perpetuity. It is made available under aCC-BY 4.0 International license.

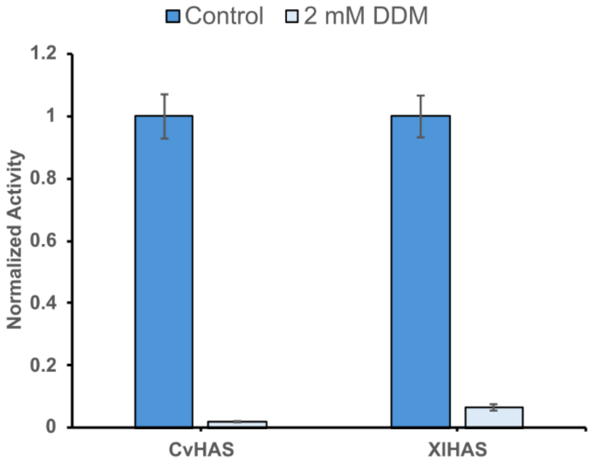

Supplementary Figure 6: HA synthesis activity is abolished by DDM. Measurements of HAS activity were taken as the average of three technical replicates. Activity values for CvHAS and XlHAS were independently normalized to the control condition. Error bars correspond to the standard deviation from the mean.

bioRxiv preprint doi: https://doi.org/10.1101/2025.10.17.683186; this version posted October 18, 2025. The copyright holder for this preprint (which was not certified by peer review) is the author/funder, who has granted bioRxiv a license to display the preprint in perpetuity. It is made available under aCC-BY 4.0 International license.

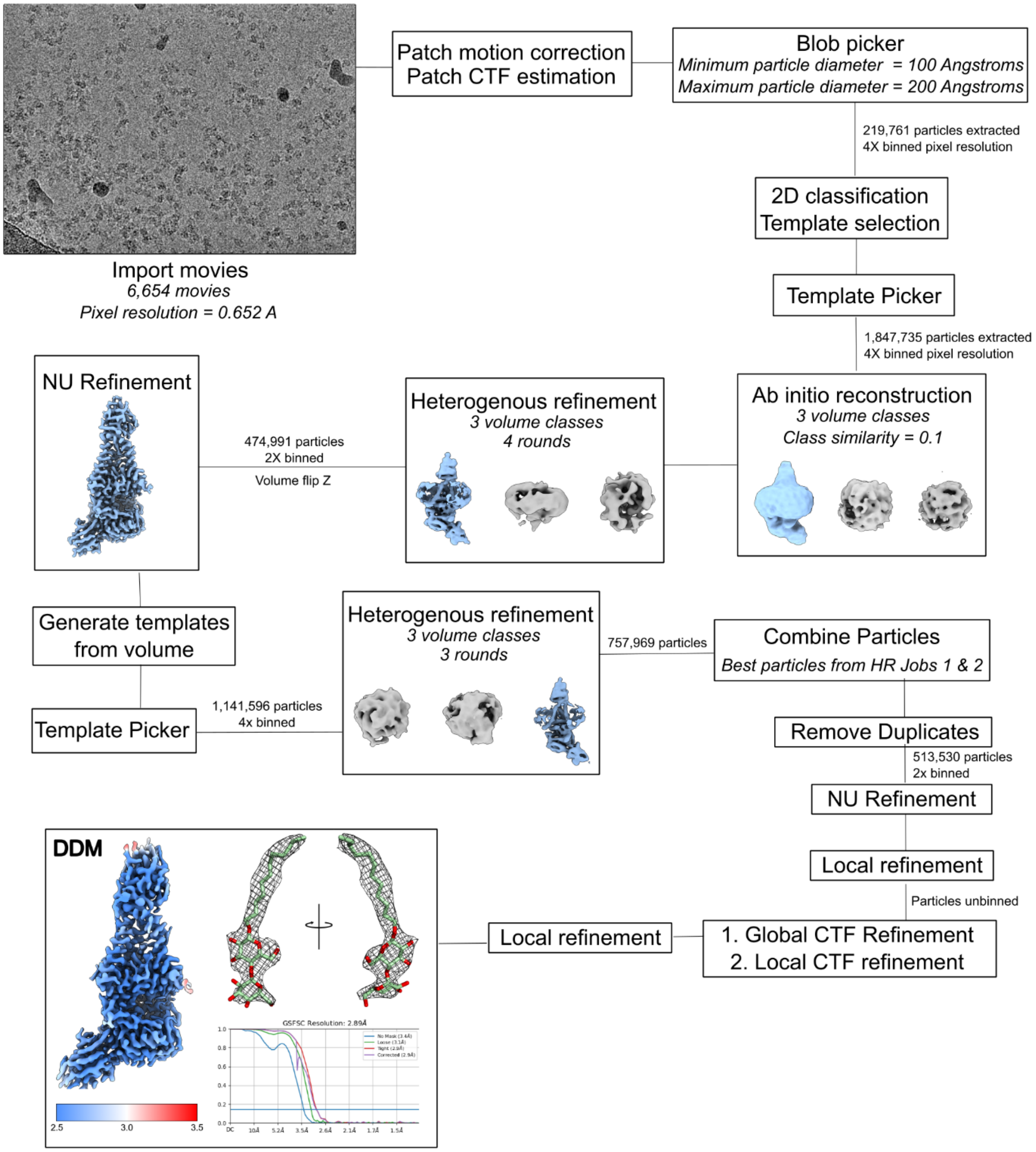

Supplementary Figure 7: CryoEM Data processing for DDM bound CvHAS. CryoSPARC data processing workflow for CvHAS bound to DDM. Carves of DDM density are shown as a black mesh. Local resolution estimates calculated at FSC = 0.143 are reported in Å.

bioRxiv preprint doi: https://doi.org/10.1101/2025.10.17.683186; this version posted October 18, 2025. The copyright holder for this preprint (which was not certified by peer review) is the author/funder, who has granted bioRxiv a license to display the preprint in perpetuity. It is made available under aCC-BY 4.0 International license.

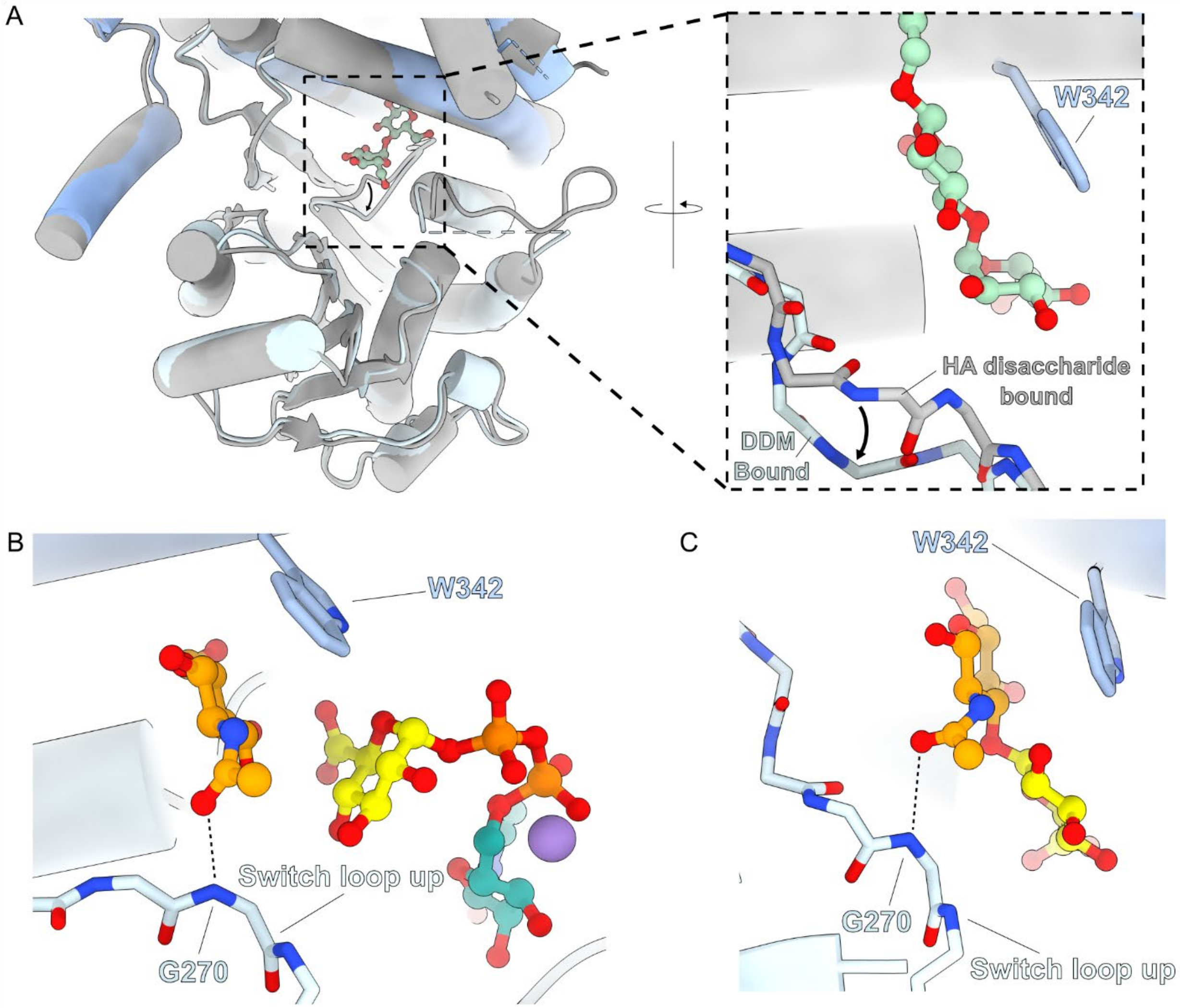

Supplementary Figure 8: Switch loop movement in GlcNAc, HA disaccharide and DDM-bound CvHAS structures. A, Superimposed structures for the GlcNAc primed, UDP-GlcA-bound CvHAS (grey) and DDM-bound CvHAS (blue). Switch loop movement is indicated by a black arrow. B-C, Position of the switch loop and interactions with GlcNAc in primed, UDP-GlcA-bound (PDB ID: 8snd) CvHAS (B) and HA disaccharide-bound (PDB ID: 8snc) CvHAS (C).

bioRxiv preprint doi: https://doi.org/10.1101/2025.10.17.683186; this version posted October 18, 2025. The copyright holder for this preprint (which was not certified by peer review) is the author/funder, who has granted bioRxiv a license to display the preprint in perpetuity. It is made available under aCC-BY 4.0 International license.

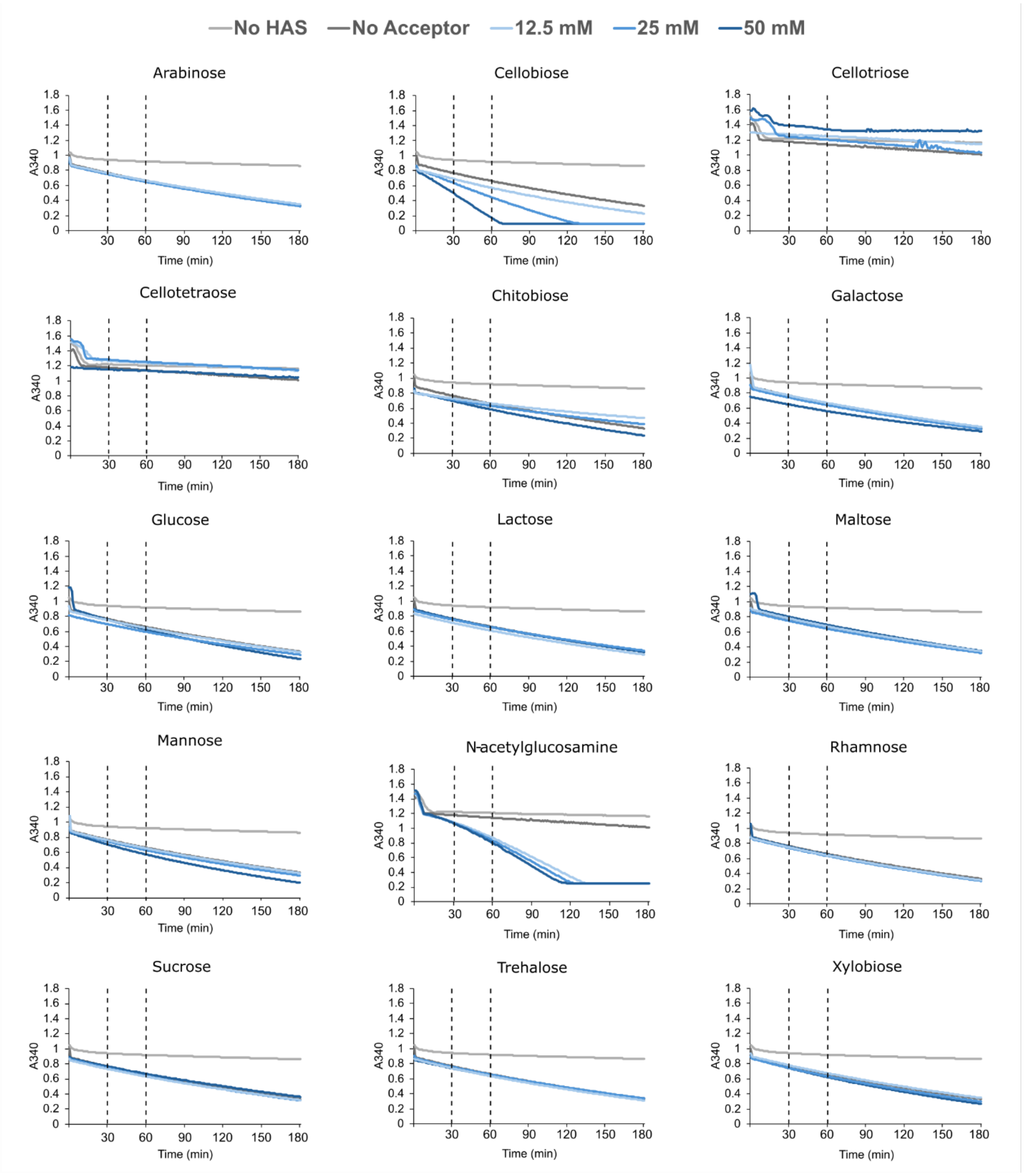

Supplementary Figure 9: Kinetic traces of UDP-GlcA hydrolysis. Individual kinetic traces for UDP-GlcA turnover in the presence of potential non-canonical glycosyl transfer acceptors supplemented at 12.5, 25, and 50 mM concentrations. The window used for determining reaction velocities is indicated by two vertical dashed lines.

bioRxiv preprint doi: https://doi.org/10.1101/2025.10.17.683186; this version posted October 18, 2025. The copyright holder for this preprint (which was not certified by peer review) is the author/funder, who has granted bioRxiv a license to display the preprint in perpetuity. It is made available under aCC-BY 4.0 International license.

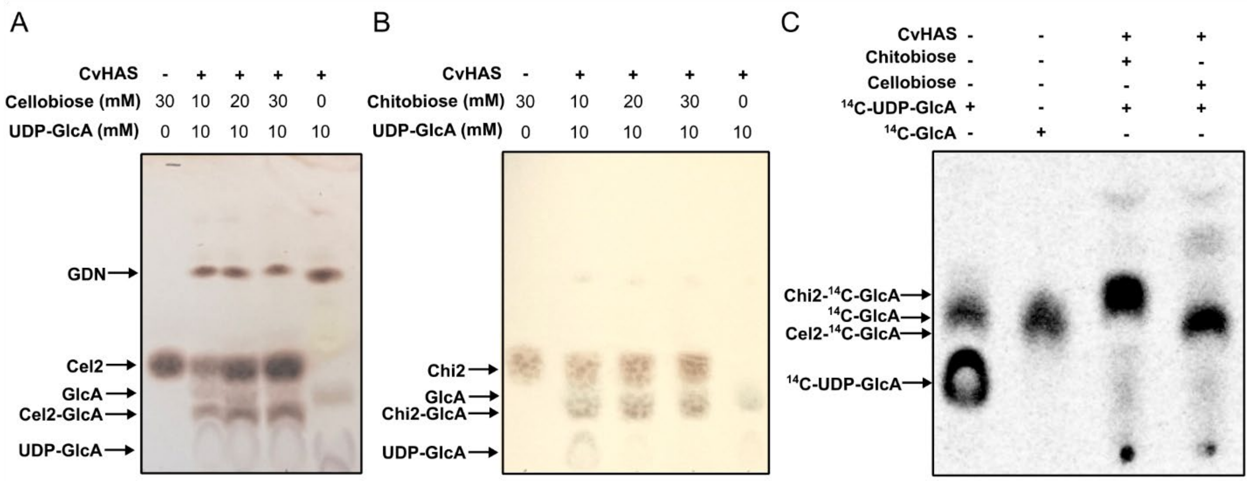

Supplementary Figure 10: Extension of cellobiose and chitobiose by GlcA. A, TLC analysis of cellobiose (Cel2) titration in the presence of excess UDP-GlcA and CvHAS. Staining was performed with thymol reagent. B, TLC analysis of chitobiose (Chi2) titration. In the presence of excess UDP-GlcA and CvHAS. Staining was performed with diphenylamine reagent. C, Autoradiograph of TLC experiment measuring transfer of ^{14}C-GlcA to cellobiose and chitobiose.

bioRxiv preprint doi: https://doi.org/10.1101/2025.10.17.683186; this version posted October 18, 2025. The copyright holder for this preprint (which was not certified by peer review) is the author/funder, who has granted bioRxiv a license to display the preprint in perpetuity. It is made available under aCC-BY 4.0 International license.

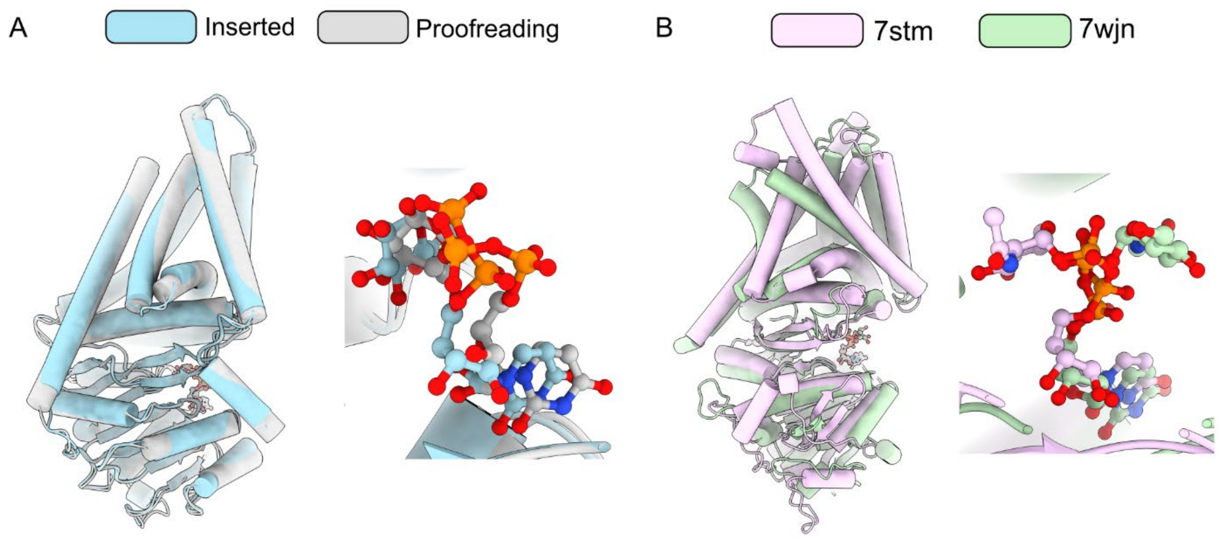

Supplementary Figure 11: Comparison of UDP-GlcA binding by CvHAS to UDP-GlcNAc binding by CHS. A, Inserted (light cyan) and proofreading (light grey) UDP-GlcA-bound CvHAS structures superimposed. B, Structures of *C. albicans* CHS-2 (PDB ID: 7stm, light purple) and *P. sojae* CHS-1 (PDB ID: 7wjn, light green) bound to UDP-GlcNAc superimposed. 7stm corresponds to the proposed 'inserted' UDP-GlcNAc pose for CHS.
